# Association of maternal exposure to endocrine disruptor chemicals with cardio-metabolic risk factors in children during childhood: a systematic review and meta-analysis of cohort studies

**DOI:** 10.1186/s13098-024-01320-0

**Published:** 2024-04-04

**Authors:** Mehran Rahimlou, Mir Ali Mousavi, Hossein Chiti, Mazyar Peyda, Seyedeh Neda Mousavi

**Affiliations:** 1 Department of Nutrition, School of Public Health, Zanjan University of Medical Sciences, Zanjan, Iran; 2 Department of General Surgery, Ayatollah Mousavi Hospital, Zanjan University of Medical Sciences, Zanjan, Iran; 3 Zanjan Metabolic Diseases Research Center, Zanjan University of Medical Sciences, Zanjan, Iran; 4 Department of Environmental Health Engineering, School of Public Health, Zanjan University of Medical Sciences, Honarestan St., Janbazan St., Zanjan, Iran

**Keywords:** Metabolic disorders, Endocrine disruptor chemicals, Systematic review, Meta-analysis

## Abstract

**Background:**

In the present systematic review and meta-analysis, the association of maternal exposure to the endocrine disrupting chemicals (EDCs) with cardio-metabolic risk factors in children during childhood for the first time.

**Method:**

The PubMed, Scopus, EMBASE, and Web of Science databases were systematically searched, up to Feb 2023. In total 30 cohort studies had our inclusion criteria. A random-effects model was used for the variables that had considerable heterogeneity between studies. The Newcastle–Ottawa Scale (NOS) tool was used to classify the quality score of studies. All statistical analyses were conducted using Stata 14 and P-value < 0.05 considered as a significant level.

**Results:**

In the meta-analysis, maternal exposure to the EDCs was weakly associated with higher SBP (Fisher_Z: 0.06, CI: 0.04, 0.08), BMI (Fisher_Z: 0.07, CI: 0.06, 0.08), and WC (Fisher_Z: 0.06, CI: 0.03, 0.08) z-scores in children. A significant linear association was found between maternal exposure to the bisphenol-A and pesticides with BMI and WC z-score in children (p < 0.001). Subgroup analysis showed significant linear association of BPA and pesticides, in the urine samples of mothers at the first trimester of pregnancy, with BMI and WC z-score in children from 2–8 years (p < 0.05).

**Conclusion:**

Prenatal exposure to the EDCs in the uterine period could increase the risk of obesity in children. Maternal exposure to bisphenol-A and pesticides showed the strongest association with the obesity, especially visceral form, in the next generation.

**Supplementary Information:**

The online version contains supplementary material available at 10.1186/s13098-024-01320-0.

## Introduction

Endocrine disruptors are defined as the exogenous chemicals that interfere with any aspect of hormone action [1]. Endocrine disruptor chemicals (EDCs) are natural and manufactured compounds that are found in pesticides, metals, plastic bottles, food containers, detergents, flame-retardants, toys, and cosmetics [1, 2]. Daily exposure to these materials is an inseparable part of contemporary life. Human are constantly exposed to these chemicals in food, air, and water. Industrial and agricultural toxins, including dioxins, perchlorates, organochlorines, organophosphates, and carbamates, are some the natural examples of EDCs. Bisphenol-A (BPA), phthalates, diethylstilbestrol and parabens are residential part of manufactured substances [3–7]. Regarding the importance of the developmental origin of health and disease theory, exposure to these exogenous chemicals during pregnancy predisposes the fetus to organ dysfunction and chronic diseases in adulthood because of long-lasting and permanent alterations in the molecular, cellular, and hormonal signaling pathways [8–12]. Identifying the most important and dangerous EDCs, and initiating preventive strategies will help to minimize the health and economic consequences of EDCs for future generations. Herein, the associations between maternal exposure to EDCs during pregnancy with the mean changes of factors related to the cardio-metabolic disorders including triglyceride (TG), total cholesterol (TC), low density lipoprotein- cholesterol (LDL-C), high density lipoprotein-cholesterol (HDL-C), fasting blood sugar (FBS), systolic and diastolic blood pressure (SBP and DBP), body mass index (BMI), and waist circumference (WC) was studied in children.

## Materials and methods

### Literature search

The PubMed, Scopus, EMBASE, and Web of Science databases were systematically searched by two independent researchers for relevant studies published before February 2023. Detailed search strategies have been presented in Additional file 1: Table S1. There was not any language restriction. The search was performed using search terms, that were based on a combination of indexed and free-text terms included: “endocrine-disrupting,” endocrine disruptor, endocrine-disrupting chemicals, maternal, pregnancy, "maternal exposure" "lipid profile," triglyceride, "low-density lipoprotein," high-density lipoprotein," LDL-C, HDL-C, cholesterol, "blood pressure," "systolic blood pressure," "diastolic blood pressure," "fasting blood sugar," FBS, weight, BMI, body mass index, and waist circumference. To avoid losing an article, references of all review articles were also checked manually. The reference list of the included articles was also manually searched.

### Study selection

Inclusion criteria were based on the population, exposure, comparison, outcome and study design (PECOS) approach (11) as follows: (1) population: pregnant women and child, (2) exposure: exposure to EDCs during the pregnancy, (3) comparison: pregnant women with the higher degrees of exposure versus the lower degrees, (4) outcome: estimated changes in TG, TC, LDL-C, HDL-C, FBS, SBP, DBP, BMI and WC, (5) study design: cohort studies.

We evaluated studies based on the following inclusion criteria: studies with cohort design that examined the association between EDC exposure in pregnancy and its effects on a child’s outcome that have been reported as an odds ratio (OR) with a 95% confidence interval (CI) or could be calculated from the provided data. We excluded studies if they had the following criteria: case–control, and cross-sectional design, reviews, letters, interventions, and conference papers, publications with no complete reports, exposure assessment in children only. In the case of studies in which more than one article was published from the same data, a study with a larger sample size was included in the meta-analysis.

### Data extraction and qualitative assessment

At the first, descriptive information was extracted from all studies. This information included the name of the first author, year of publication, the country of study, type of EDC, sample size, the mean age of participants, and the risk estimates with 95% CI. The Newcastle–Ottawa Scale (NOS) tool was used to classify the quality score of studies as follows: low quality = 0–4; moderate-quality = 5–6; high quality = 7–9 [13]. Two reviewers independently conducted the risk of bias assessment (MR and SNM); disagreements were resolved after discussion with a third reviewer (MAM).

### Statistical analysis

All statistical analyses were conducted using Stata 14 (Stata Corporation, College Station, TX, U.S.A.). We aggregated the studies into four general groups according to the type of EDC including BPA, pesticides, phthalates, and other EDCs. A fixed- and random- model effects were used to estimate the effect size and heterogeneity of studies. The heterogeneity was evaluated by the χ2-Q statistics and I^2^ that is classified as follows: I^2^ < 30% mild, I^2^ = 30–75% moderate and high if I^2^ > 75% [14]. A random-effects model was used for the variables that had considerable heterogeneity between studies. To identify the potential sources of heterogeneity, the subgroup analysis was performed based on the country (the USA vs. other countries), sample type (serum or urine), pregnancy time for sampling (the first trimester of pregnancy vs. 2nd or 3th trimesters), age of child in the evaluation time (≤ 4 or > 4 yrs.), and the type of EDC (BPA, phthalates, pesticides or other EDC). The effect of each study on the overall estimates was studied by the leave-one-out method. Also, the publication bias was assessed by the funnel plots, Begg's rank correlation, and Egger's linear regression tests. We followed the conventional cut-offs to interpret the effect size (r) as weak (≤ 0.35), moderate (≤ 0.36—0.67), and strong (≤ 0.68–1.00) [15].

## Results

### Findings of the systematic review

**Fig. 1:**
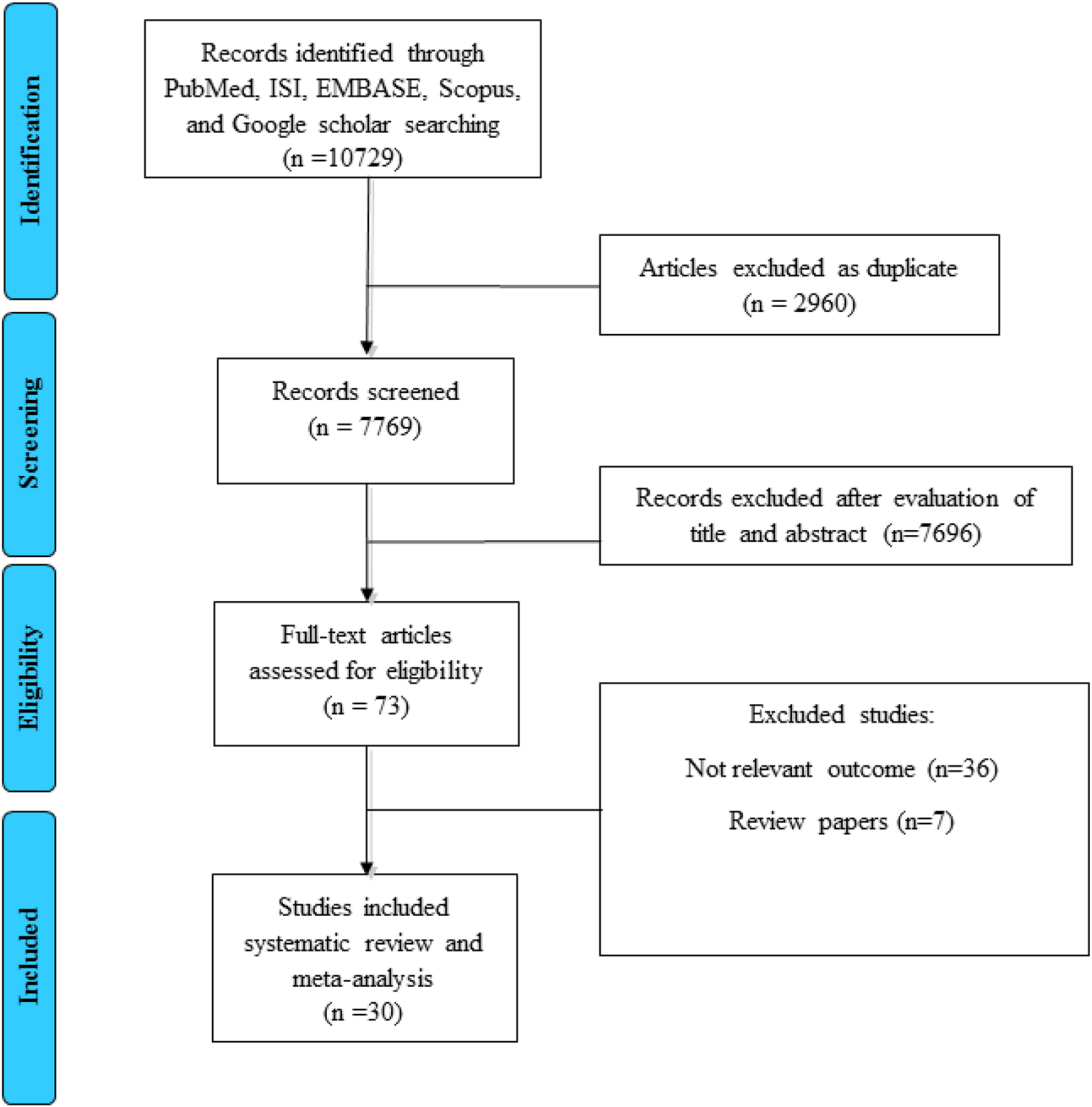
PRISMA flow diagram of study selection

As shown in Fig. 1**,** the early search resulted in 7769 studies after duplicate removal. After the first screening and reading the article’s title and abstract, 6480 papers were excluded due to the unrelated titles. In the second phase of screening, 1216 articles were again excluded due to the unrelated data, animal subjects, being review articles, etc. In total, 73 papers were evaluated for eligibility, and 43 articles were excluded for the following reasons: maternal exposure was not assessed, the concentration of chemicals was not reported, risk estimate was not reported, and two studies were based on the same data. Finally, 30 studies were included for the final analysis [16–45].

The characteristics of the included studies in this systematic review and meta-analysis have been presented in Table 1. These studies were published among 2008 to 2022, and all included studies had a cohort design. Among the included studies, ten studies were reported from the USA and twenty from other countries. Studies included in the final analysis were conducted in several countries, including: Spain [18, 20, 24, 29, 33, 35], China [22, 44], USA [17, 19, 26, 27, 31, 36, 40, 42], Italy [45], Netherland [21], Denmark [23, 39], Korea [25, 30, 43], Belgium [41], Canada [32, 34], and Greece [28, 29]. Except of one cohort study that evaluated prenatal exposure to the EDCs in newborns, other studies assessed this association in the age of 2 to 8 years. The number of cases varied from 105 to 4065. The type of evaluated EDCs was different in most studies. As mentioned, we categorized and evaluated EDCs into four main groups, including BPA, phthalates, pesticides, and other EDCs. Based on this category, eleven studies evaluated BPA, twelve studies assessed phthalates, ten evaluated pesticides, and finally, eleven cohorts examined other EDCs. It should be noted that some studies have examined more than one EDC. In terms of the study quality, the total score quality was shown in Table 1**,** and the detailed score for each study was reported in Additional file 1: Table S2. According to the total score, nine studies had moderate-quality, and others had good quality.

**Table 1:** Characteristics of the included studies

Author, Year	Country	Total/ subclasses of EDCs	Mother age, yrs (mean ± SD)	Pre-preg BMI (kg/m^2^)	Sample size (F/M)	Outcome	Sample	Adjustments/match	Total quality score
Montazeri, 2022	Spain	BPA, Phthalate, phenols	31.9 ± 4.1	23.8 ± 4.5	150/155	BP	Spot urine	For child age and height at visit, child sex, mother’s age at delivery, pre-pregnancy BMI, gestational weight gain, social class, parental cardiovascular history, smoking during pregnancy and gestational age	7
Zuo, 2022	USA	BPA	48 ± 17.75	NM	4065/3831	BMI	Spot urine	Age, urinary creatinine, marital status, total energy intake, race/ethnicity, education, ratio of family income to poverty, smoking, drinking, and exercise	9
Yang, 2021	China	Pesticide	28.2 ± 3.2	NM	495/544	BMI	Cord	Infant gender, maternal age, maternal education, pre-pregnancy BMI, pregnancy weight gain, maternal height, parity, passive smoking, and duration of breastfeeding	7
							Serum		
Güil-Oumrait, 2021	Spain	POPs	29.84 ± 4.48	NM	187/192	WC	Cord serum	Maternal characteristics (i.e. parity history, pre-pregnancy BMI, education, socioeconomic status, smoking, and age at pregnancy), and child’s follow up visit. Body fat % model was additionally adjusted by sex	7
						BMI			
						BP			
Berger, 2021	USA	MBzP, MEP, MBP, MiBP MCPP, MCOP, MCNP, DEHP	26.7 ± 5.3	NM	Total: 309	BMI	Urine	Age at delivery, maternal education, years lived in the U.S. at delivery, poverty status during pregnancy (at or below vs. above poverty threshold), and the childhood frequency of fast-food intake at age five (< 1 time per week, 1–2 times per week, and 3 + times per week)	7
Kupsco, 2020	USA	Phthalates	27.9 ± 5.7	26.4 ± 4.1	235/228	TG	Serum	Phthalate metabolite concentrations are vary throughout pregnancy, and from day to day and throughout a 24-h period	5
						TC			
						HDL.C			
Jensen, 2020	Denmark	BPA, POPs, PFOS, PFHxS, PFOA, PFDA, PFNA	30.2 ± 4.5	NM	275/318	WC	Serum	Maternal age, parity, pre-pregnancy BMI, educational level, smoking, sex, and adiposity marker at birth	6
						BMI			
						TG			
						TC			
						LDL-C			
						HDL-C			
Warner, 2020	Italy	Dioxins	27.8 ± 4.8	NM	222/204	TG	Serum	Age at interview, sex, primary wage earner education, and maternal age at pregnanc	7
						TC			
						LDL-C			
						HDL-C			
						FBS			
Sol, 2020	Netherland	Phthalate	30.9 ± 4.6	22.7 (18.5–34.9)	526/538	BP	Urine	Child’s age and standardized height and maternal age, education, parity, ethnicity, pre-pregnancy body mass index, alcohol consumption and smoking habits (specifically in early, mid and late pregnancy)	6
Ouyang, 2020	China	BPA	30.6 ± 3.5	21.7 ± 3.3	105/113	BP, FBS	Urine	(1) Child age; (2) birthweight for gestational age (LGA and non-LGA); (3) child urinary BPA (low, medium and high level in tertiles) and weight-for-length z-score; (4) maternal passive smoking (yes or no), child passive smoking (yes or no), and infant 0–6 months breastfeeding type (formula, exclusive breastfeeding, and mixed feeding)	9
Lee, 2019	Korea	DEHP, MnBP, MEHHP, MEOHP	NM	NM	226/255	BMI	Urine	Age, body mass index, household income level, and maternal education level	8
Manzano-Salgado, 2017	Spain	PFHxS, PFOA, PFNA, PFOS,	31.5 ± 5.5	NM	600/630	WC	Plasma	Maternal region of residence, country of birth, parity, pre-pregnancy BMI, previous breastfeeding, and by the age at follow-up and sex of the child	8
						BMI			
						TG			
						TC			
						LDL-C			
						HDL-C			
Bae, 2017	Korea	BPA	31.2 ± 3.6	NM	227/259	BP	Urine	Adjusted for age, sex, height, weight, birth weight, gestational age at birth, maternal age at enrollment, parental history of hypertension, father’s education, environmental tobacco smoke, duration of vigorous physical activity per week, and current infection	6
Vuong, 2016	USA	PBDEs	29.3 ± 2.7	25.7 ± 4.3	200/118	WC	Serum	Maternal age, race, education, income, maternal smoking status, maternal depression, fresh fruit and vegetable intake during pregnancy. Additionally adjusted by maternal height	7
						BMI			
Maresca, 2016	USA	Phthalate, MCPP, MiBP, MBP, MBzP	28.5 ± 3.4	NM	181/156	WC	Urine	Age (in months) at time of measurement, maternal pre-pregnancy obesity, birth weight, maternal race/ethnicity, receipt of public assistance during pregnancy, and urinary specific gravity. Metabolite concentrations	8
						BMI		Were ln-transformed for analyses	
Vafeiadi, 2016	Spain	BPA	29.4 ± 5.1	24.4 ± 4.9	219/275	WC	Urine, Serum	Maternal educational level, maternal age, pre-pregnancy BMI, working status during pregnancy, child sex, Z-score of birth weight for gestational age and breastfeeding status	6
						BMI			
						TG			
						TC			
						HDL-C			
						BP			
Buckley, 2016	USA	MEP, MnBP, MiBP, MCPP, MBzP, DEHP	NM	NM	129/126	BMI	Urine	Adjusted for cohort, maternal race/ethnicity, maternal age at delivery, maternal education, maternal work status during pregnancy, maternal pre- pregnancy BMI, maternal height, gestational weight gain, maternal smoking during pregnancy, natural log creatinine, calendar date of urine collection, parity, child’s sex, breastfeeding, and months of age at follow-up	9
Braun, 2016	Canada	PFOA, PFNA, PFHxS, PFOS	29 ± 5.9	NM	110/175	WC	Serum	Maternal age, race, education, income, parity, employment, marital status, depressive symptoms, BMI at 16 weeks gestation, fruit/vegetable consumption, fish consumption, prenatal vitamin use, and maternal serum cotinine concentrations. The waist circumference model is also adjusted for child age in months	6
						BMI			
Vafeiadi, 2015	Greece	PCBs, DDE, HCB	29.9 ± 5	24.4 ± 4.7	331/358	BMI	Serum	Maternal variables: triglycerides and cholesterol, age, BMI before pregnancy, parity, educational level, smoking during pregnancy	7
						WC		Variables of the child: weight at birth, sex, lactation, gestational age and exact age at the time of measurement	
						BP			
Kim, 2015	Korea	diethylhexyl phthalate	33.5 ± 4.5	NM	65/63	TG	Urine, umbilical cord blood samples	NM	6
						TC			
Erkin-Cakma, 2015	Canada	PBDEs	27.5 ± 5	NM	125/99	WC	Serum	Maternal age, education, pre-pregnancy BMI, years lived in the United States, gestational weight gain, poverty during pregnancy; and child gestational age, duration of breastfeeding, and fast food and soda consumption at age 7	7
						BMI			
Valvi, 2015	Spain	HMWPm, LMWPm	39.7 ± 1.4	NM	186/205	WC	Urine	adjusted for child sex, exact age at examination, and maternal characteristics (country of origin, age at delivery, parity, education, social class, pre-pregnancy BMI, and smoking in pregnancy	7
						BMI			
						BP			
Agay-Shay, 2014	Spain	HCB, MEP, MnBP, MiBP, MBzP, MEcPP, MEHHP, DDE, BPA	NM	NM	total: 470	BMI	Urine	child’s sex (male, female), gestational age (continuous in weeks), birth weight (continuous, in grams), exact age at the time that the outcome was measured (continuous, in months), and maternal country of origin (Spain, non-Spain), maternal age at delivery (continuous, in years), maternal pre-pregnancy BMI (continuous, in kilograms per meter squared), maternal weight gain during pregnancy (low, recommended, or high)	9
Tang-Peronard, 2014	USA	PCBs, DDE	27.1 ± 4.7	23.9 ± 3.9	271/290	WC	Serum	NM	8
						BMI			
Warner, 2014	USA	o,p-DDT, p,p-DDT, p,p-DDE	26.1 ± 5	NM	118/143	WC	Serum	Maternal pre-pregnancy BMI, birth weight, and child television time	8
						BMI			
Harlely, 2013	USA	BPA	26 ± 4.5	NM	167/123	WC	Urine	Maternal pre-pregnancy BMI, household income, maternal education level, maternal years of residence in the United States, smoking during pregnancy, soda consumption during pregnancy, and child’s fast food and sweet consumption at age 9 year	8
						BMI			
Andersen, 2013	Denmark	PFOS, PFOA	30.6 ± 6.2	22.9 ± 4.7	387/400	WC	Serum	Including child’s age, maternal age, parity, pre-pregnancy BMI, smoking, socioeconomic status, and gestational age at blood drawing	9
						BMI			
Cupul-Uicab, 2013	USA	HCH, p,p-DDT, HCB, PCBs, p,p-DDE	NM	NM	641/1042	BMI	Serum	(1)Total cholesterol, triglycerides, study center, mother’s race, socioeconomic index, education, smoking during pregnancy, prepregnancy BMI, and child’s sex and birth order. (2) child’s exact age at anthropometric measurements	6
Delvaux, 2013	Belgium	PCBs, HCB	30 ± 5.5	NM	57/57	WC	Serum	Total cholesterol, triglycerides, study center, mother’s race, socioeconomic index, education, and smoking during pregnancy, pre-pregnancy BMI, and child’s sex and birth order. Additionally adjusted for child’s exact age at anthropometric measurements	7
						BMI			
Smink, 2008	Spain	HCB, total PCBs	NM	NM	482	BMI	Serum	(1) Age and sex, (2) maternal age, height, pre-pregnancy over-weight or obesity, education, parity and child’s sex and current age. (3) 2 + birthweight	7

*BPA* bisphenol-A, *POPs* Persistent organic pollutants, *MBzP* monobenzyl phthalate, MEP monoethyl phthalate, *MBP* mono-n-butyl phthalate, *MiBP* mono-isobutyl phthalate, *MCPP* mono-(3-carboxypropyl) phthalate, *MCOP* Mono-carboxy-isooctyl phthalate, *MCNP* mono-carboxyisononyl phthalate, *DEHP* three di-(2-ethylhexyl) phthalate, *PFOS* perfluorooctanesulfonic acid, PFHxS perfluorohexanesulfonic acid, *PFOA* perfluorooctanoic acid, *PFDA* perfluorodecanoic acid, PFNA perfluorononanoic acid, *MnBP* mono-n-butyl phthalate, *MEHHP* mono(2-ethyl-5-hydroxyhexyl)-phthalate, *MEOHP* Mono(2-ethyl-5-oxohexyl)-phthalate, *PBDEs* polybrominated diphenyl ethers, PCBs polychlorinated biphenyls, *DDE* Dichloro-2,2-bis (p, p’-chlorophenyl) ethylene, *HCB* hexachlorobenzene, *HMWPm* high molecular weight phtalate, *LMWPm* low molecular weight phtalate, *MEP* monoethyl phthalate, *MECPP* mono-(2-ethyl-5-carboxypentyl) phthalate, *DDT* 1,1,1-trichloro-2,2-bis (p-chlorophenyl)-ethane, *HCH* hexachlorocyclohexane, BMI body mass index, *WC* waist circumference, *BP* blood pressure, *TG* triglyceride, *TC* total cholesterol, *FBS* fasting blood sugar, *HDL-C* high density lipoprotein-cholesterol, *LDL-C* low density lipoprotein-cholesterol

### Association between maternal exposures to the EDCs with the glycemic profile

Only two studies evaluated the effects of maternal EDC exposure with child’s FBS [22, 45]. Warner et al. included 426 children in their study and evaluated the association between maternal 2,3,7,8-tetrachlorodibenzo-p-dioxin (TCDD) and glycemic profile in children. Maternal exposure to the TCDD showed an inverse association with serum insulin (adj-β = -1.24 μIU/mL, 95% confidence interval (CI): -2.38, -0.09) and HOMA2-B (adj-β = − 10.2% decrease, 95% CI: − 17.8, − 1.9) in girls, but these associations were not statistically significant among boys (insulin: adj-β = 0.57 μIU/mL, 95% CI: − 0.84, 1.98, P for interaction = 0.04; and HOMA2-B: adj-β = 0.8% increase, 95% CI -10.7, 13.9, P for interaction = 0.11) [45]. In addition, medium maternal prenatal BPA level showed a moderately significant association with serum plasma glucose in boys 0.36 (95% CI: 0.04 to 0.68) in another study. However, no associations were found between prenatal exposure to BPA and serum insulin level in girls and boys [22].

### Association between maternal exposures to EDCs with lipid profile

**Fig. 2:**
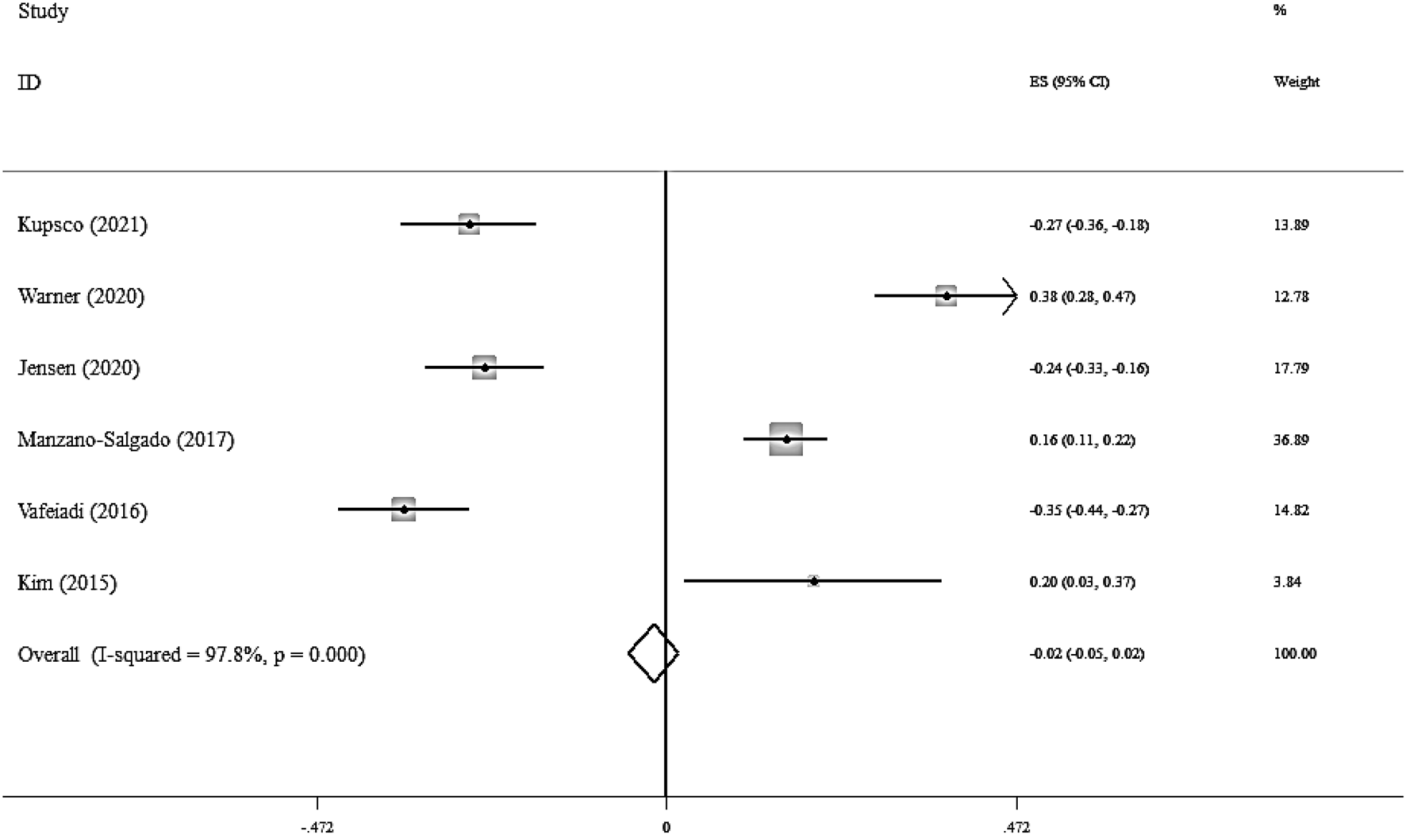
Overall effect of maternal exposure to the EDCs on serum triglyceride level in children

Overall, six studies considered the association between the maternal exposure to the EDCs and serum TG level [19, 23, 24, 29, 30, 37]. These studies included 3334 participants. No significant association was seen between the maternal exposure to the EDCs and serum TG level (Fisher_Z: -0.02, CI: -0.05, 0.02) per doubling EDCs levels (Fig. 2).

There was a high heterogeneity between studies (I^2^ = 97.8%, P < 0.001). The results of the subgroup analysis have been reported in Additional file 1: Table S3.

In a subgroup analysis, a significant association was found between maternal exposure to the EDCs and serum TG level in children, in studies performed at the second and third trimester of pregnancy. The visual inspection of the funnel plot has been presented in Additional file 1: Fig S1. The sensitivity analysis did not show any change in the results.

**Fig. 3:**
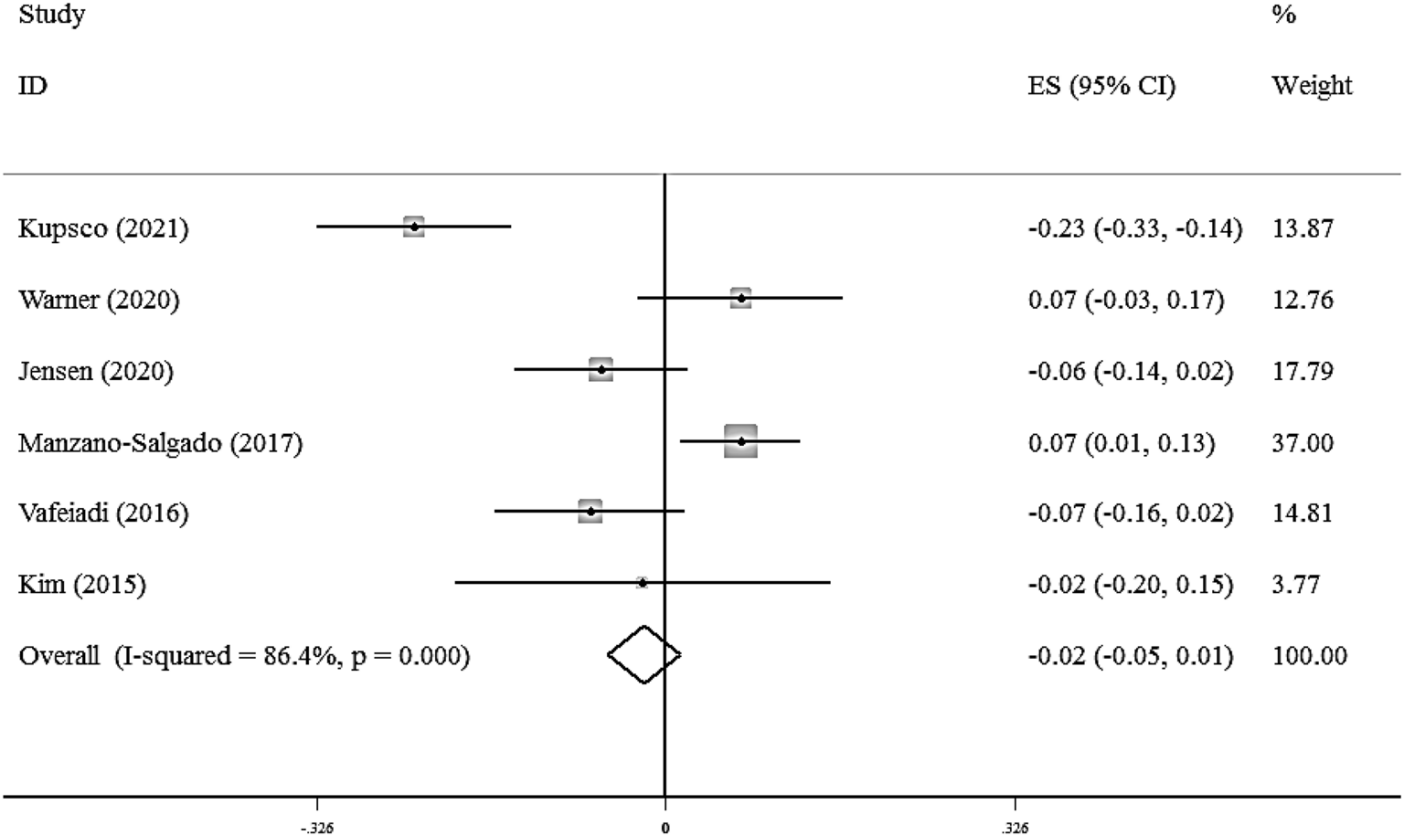
Overall effect of maternal exposure to the EDCs on serum total cholesterol level in children

There was not a significant association between maternal exposure to the EDCs with serum TC (Fisher_Z: -0.02, CI: -0.05, 0.01). A significant heterogeneity was observed among studies (I^2^ = 86.4%, P < 0·001) (Fig. 3).

Subgroup analysis did not find any source of heterogeneity regarding serum TC. Also, there was not any evidence of publication bias in the Begg (P = 0.707), Egger's regression tests (P = 0.436) and the funnel plot (Additional file 1: Fig S2). Sensitivity analysis did not show any change in the results.

**Fig. 4:**
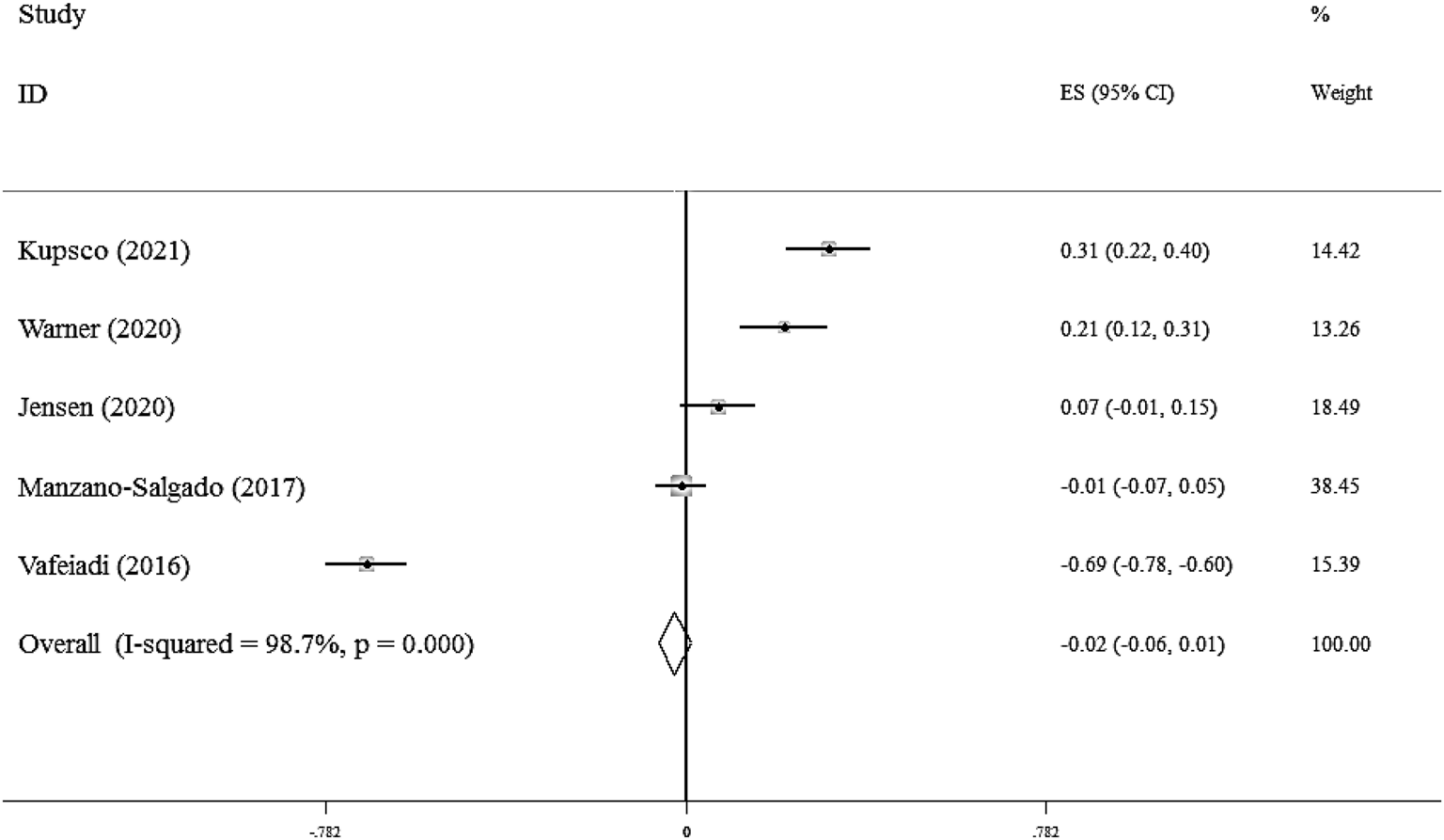
Overall effect of maternal exposure to the EDCs on serum HDL-C in children

Five studies evaluated the associations between maternal exposure to the EDCs and serum HDL.C levels in children[19, 23, 24, 28, 37]. As shown in Fig. 4, there was not any significant association between EDCs and serum HDL.C (Fisher_Z: -0.02, CI: -0.06, 0.01). Studies showed a significant heterogeneity (I^2^ = 98.7%, P < 0·001). However, in the subgroup analysis, we found a significant association in each subgroup (Additional file 1**:** Table S4).

We did not find any evidence of publication bias according to the Begg (P = 0.462), and Egger's regression tests (P = 0.990) and funnel plot (Additional file 1: Fig S3). Sensitivity analysis did not show any change in the results.

### Association between maternal exposures to the EDCs with blood pressure

**Fig. 5:**
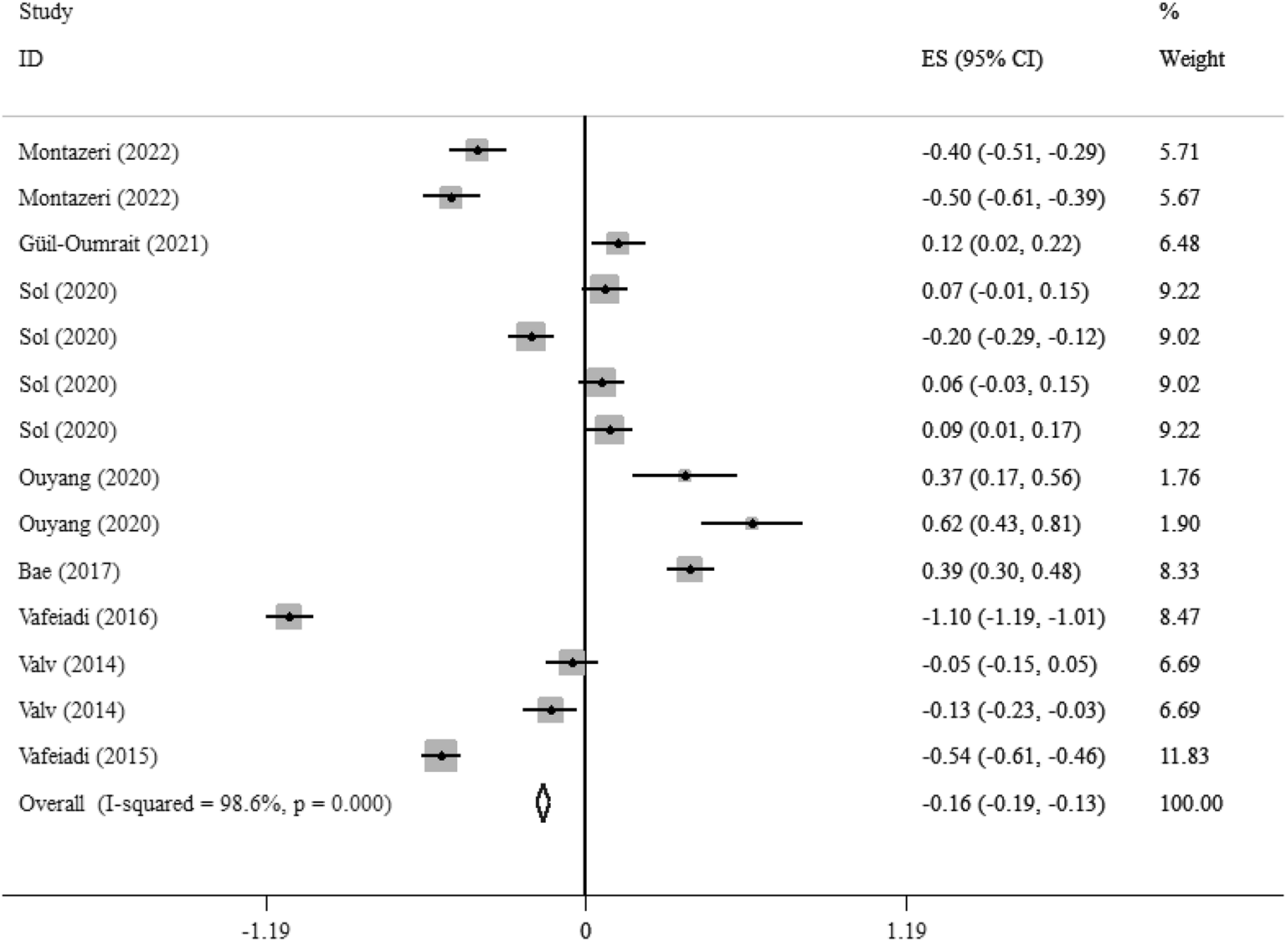
Overall effect of maternal exposures to the EDCs on DBP in children

Eight studies with 14 effect sizes reported the association between EDCs exposure with DBP [16, 18, 21, 22, 25, 28, 29, 33]. We found that EDCs exposure during pregnancy had a weakly significant correlation with lower DBP in children (Fisher_Z: -0.16, CI: -0.19, -0.13), with a significant heterogeneity between studies (I^2^ = 98.6%, P < 0·001) (Fig. 5). Subgroup analysis did not report any new findings (Additional file 1:Table 5). We did not find the source of heterogeneity in the subgroup analysis. We did not find any evidence of publication bias according to the Begg (P = 0.869), Egger's regression tests (P = 0.3), and funnel plot in term of DBP (Additional file 1: Fig S4).

**Fig. 6:**
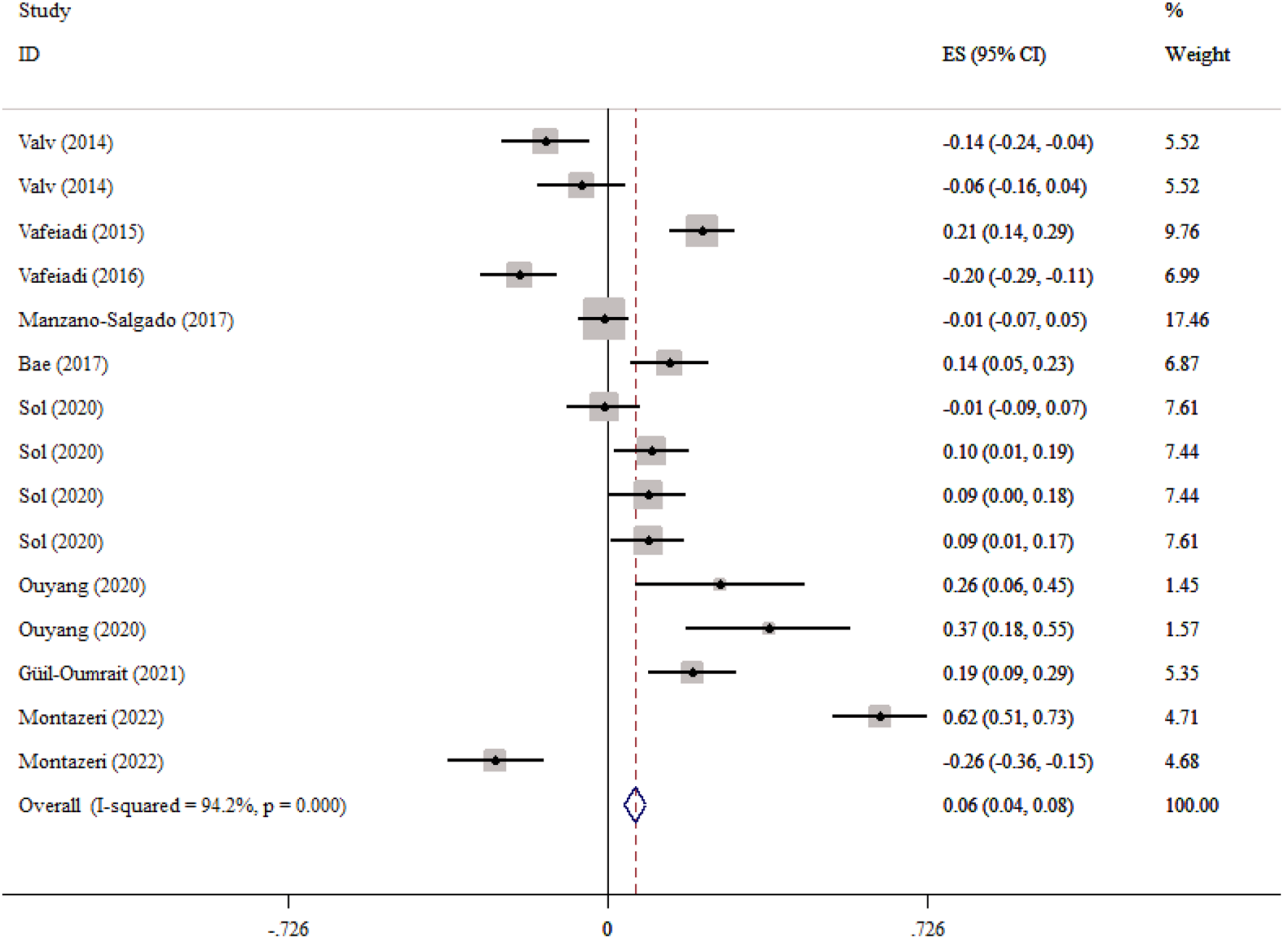
Overall effect of maternal exposures to the EDCs on SBP in children

Also, in eight studies with 15 effect sizes, the association between maternal exposure to the EDCs and SBP was reported. We found that maternal exposures to the EDCs had a weakly significant correlation with higher child’s SBP (Fisher_Z: 0.06, CI: 0.04, 0.08) (Fig. 6). The studies showed a significant heterogeneity (I^2^ = 94.2%, P < 0·001) (Fig. 5). Subgroup analysis did not find the source of the heterogeneity. We did not find any evidence of publication bias according to the Begg (P = 0.456), Egger's regression tests (P = 0.385) and the funnel plot (Additional file 1: Fig S4). Sensitivity analysis did not show any change in the results.

### Association between maternal exposures to the EDCs with child’s BMI and WC z-score

**Fig. 7:**
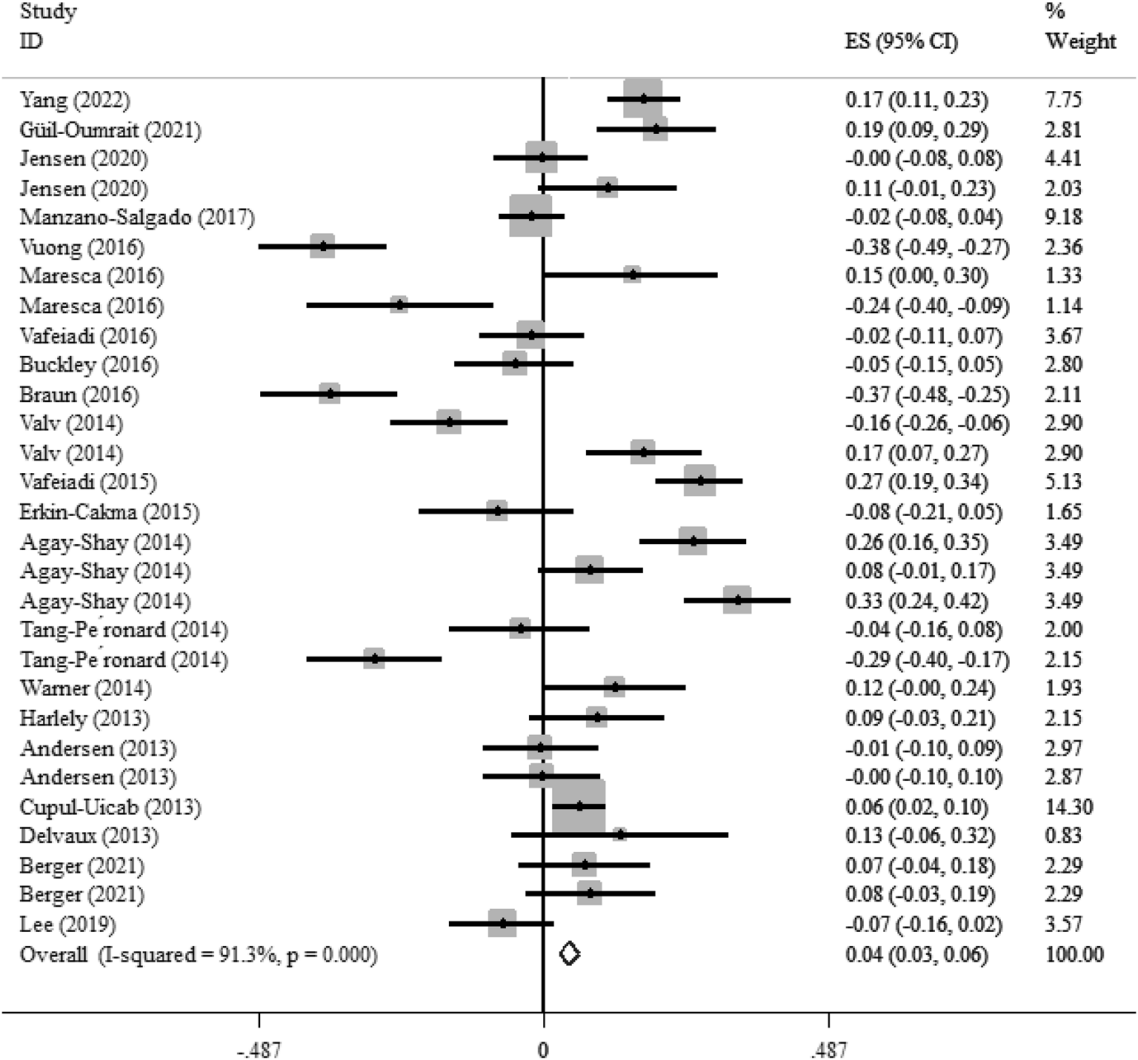
Overall effect of maternal exposures to the EDCs on BMI z-score in children

Twenty-one studies with 29 effect sizes reported the association between maternal exposures to the EDCs and child’s BMI z-score. As shown in Fig. 7, maternal exposure to the EDCs had a weakly significant correlation with higher BMI z-score in children (Fisher_Z: 0.04, CI: 0.03, 0.06). The included studies showed a significant heterogeneity (I^2^ = 91.3%, P < 0·001). Maternal exposures to the EDCs showed a significant effect on BMI z-score in the non-US countries, sampling the urine at the first trimester of pregnancy throughout all ages (P < 0.05) ( Additional file 1:Table 6). Moreover, maternal exposures to the BPA and pesticides showed a weakly significant correlation with BMI z-score in children (Fisher_Z: 0.14, CI: 0.08, 0.19, P < 0.001 for BPA and Fisher_Z: 0.1, CI: 0.08, 0.12, P < 0.001 for pesticides). Other EDCs showed a weak reverse association with BMI z-score in children (Fisher_Z: -0.09, CI: -0.13, -0.06, P < 0.001). The visual inspection of the funnel plot has been presented in Fig. 5S. Sensitivity analysis did not show any change in the results.

**Fig. 8:**
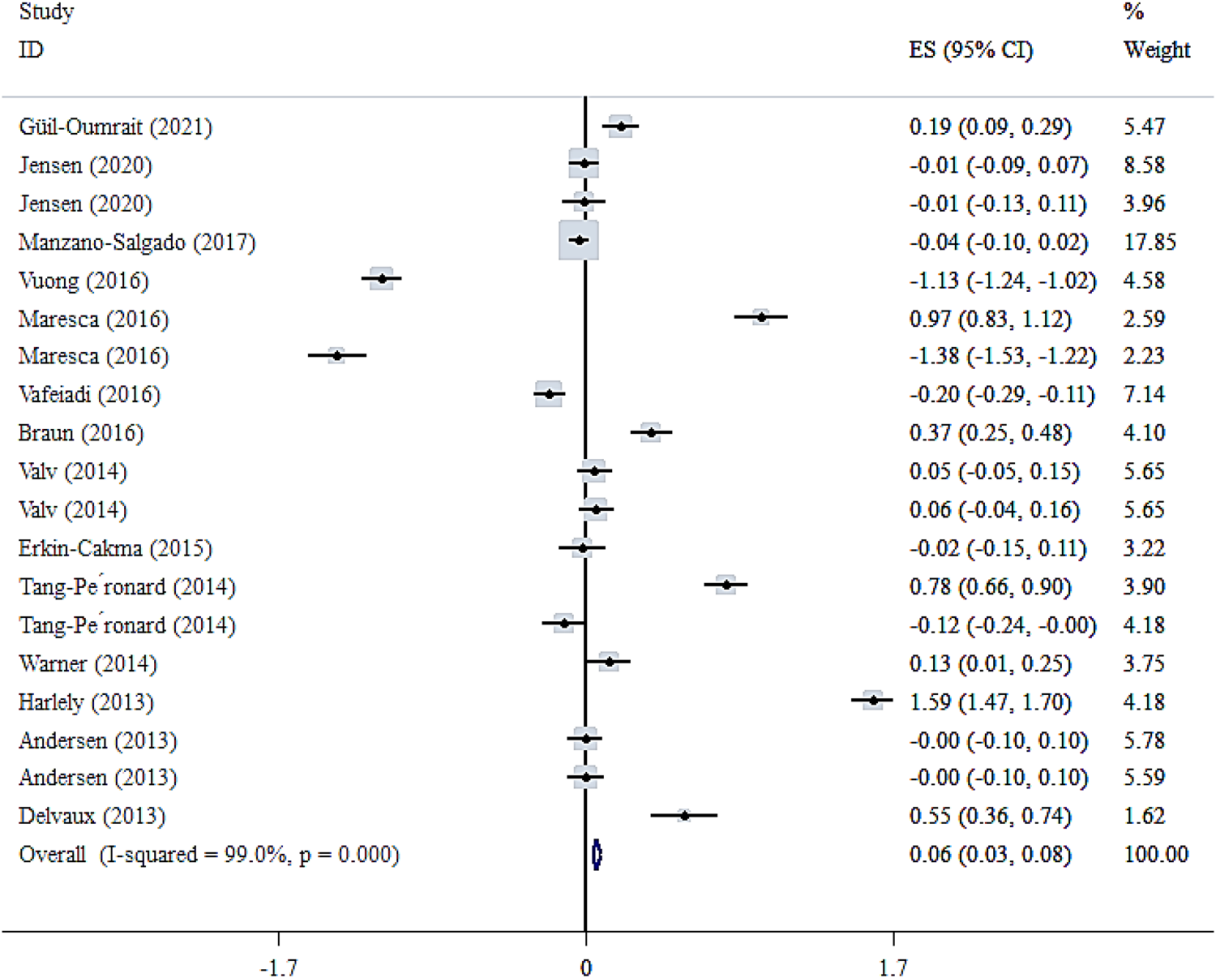
Overall effect of maternal exposures to the EDCs on WC z-score in children

Fourteen studies with nineteen effect sizes reported the association of maternal exposures to the EDCs with a child’s WC [18, 23, 24, 26–28, 32–34, 36–39, 41]. As shown in Fig. 8, there was a weakly significant association between maternal exposures to the EDCs and WC z-score in children (Fisher_Z: 0.06, CI: 0.03, 0.08). The included studies showed a significant heterogeneity (I^2^ = 99%, P < 0·001). The subgroup analysis showed a significant association between the maternal exposures to the EDC with WC z-score in the studies conducted in the USA, using urine samples in the first trimester of pregnancy and children ≤ 4. Moreover, a significant association was observed between maternal exposures to the BPA and pesticide with WC z-score. However, this association was reverse in the other EDCs (Additional file 1:Table 6). We did not find any evidence of publication bias according to the Begg (P = 0.063), Egger's regression tests (P = 0.556), and the funnel plot (Additional file 1: Fig S5). Sensitivity analysis did not show any change in the results.

## Discussion

Results of the present systematic review and meta-analysis on previous cohort studies showed a significant linear association between maternal exposures to the EDCs with BMI, WC z-scores and SBP in children. However, our results showed an inverse correlation between maternal exposures to the EDCs with DBP. There wasn’t any significant association between maternal exposures to the EDCs with lipid profile.

The results regarding the association between maternal EDC exposure and glycemic profile in children were limited, with only two studies providing relevant data [46, 47]. While Warner et al. found an inverse association between maternal exposure to 2,3,7,8-tetrachlorodibenzo-p-dioxin (TCDD) and serum insulin and HOMA2-B in girls, no significant associations were observed in boys [48]. Another study reported a significant association between medium maternal prenatal BPA levels and serum plasma glucose in boys but not in girls [47]. The observed gender-specific associations between maternal EDC exposure and glycemic parameters in children in some studies could be attributed to differences in hormonal regulation and metabolic processes between boys and girls. It's well-established that sex hormones, such as estrogen and testosterone, play crucial roles in modulating glucose metabolism and insulin sensitivity [49]. Therefore, exposure to EDCs during critical periods of development, such as prenatal or early postnatal stages, may disrupt the normal hormonal milieu and contribute to dysregulation of glycemic control in a sex-specific manner.

One potential mechanism underlying the observed associations involves disruption of the endocrine system by EDCs, leading to alterations in insulin signaling pathways and glucose homeostasis. For instance, TCDD, a well-known EDC, has been shown to affect insulin secretion and sensitivity by binding to the aryl hydrocarbon receptor (AhR) and modulating downstream signaling pathways involved in glucose metabolism [50]. Similarly, BPA, another prevalent EDC, has been implicated in impairing pancreatic β-cell function and insulin sensitivity through its estrogenic activity and interference with insulin receptor signaling [51]. Furthermore, emerging evidence suggests that EDCs may exert epigenetic effects, leading to persistent alterations in gene expression patterns related to glucose metabolism and insulin sensitivity. Epigenetic modifications, such as DNA methylation and histone acetylation, can modulate the expression of key genes involved in glycemic regulation, potentially predisposing individuals to metabolic disorders later in life [52, 53]. Importantly, these epigenetic changes may exhibit sex-specific patterns due to differences in sex chromosome composition and hormonal regulation of epigenetic machinery [54–56].

In terms of lipid profile, the meta-analysis did not reveal a significant association between maternal EDC exposure during pregnancy and serum TG or TC levels in children. However, subgroup analysis identified a significant association between maternal EDC exposure and serum TG levels in studies conducted during the second and third trimesters of pregnancy. This suggests a potential window of vulnerability during later stages of gestation. Similarly, no significant association was found between maternal EDC exposure and serum HDL-C levels in children. The lack of a significant association between maternal EDC exposure during pregnancy and serum TG or TC levels in children, as indicated by the meta-analysis, suggests that the overall impact of maternal EDC exposure on lipid metabolism may be nuanced and multifactorial. However, subgroup analysis revealing a significant association between maternal EDC exposure and serum TG levels specifically in studies conducted during the second and third trimesters of pregnancy suggests a potential window of vulnerability during later stages of gestation. This observation aligns with previous evidence suggesting that fetal development during late gestation is particularly sensitive to environmental exposures, including EDCs, which may influence metabolic programming and long-term health outcomes [57–59].

One potential mechanism underlying the observed association between maternal EDC exposure and serum TG levels in children could involve disruption of lipid metabolism pathways. EDCs, such as BPA and phthalates, have been shown to interfere with lipid synthesis, transport, and metabolism through various mechanisms, including activation of nuclear receptors (e.g., peroxisome proliferator-activated receptors, PPARs) and modulation of lipid-related gene expression[60, 61]. For example, BPA exposure has been associated with dysregulation of lipogenic genes in animal models, leading to increased hepatic lipid accumulation and altered serum lipid profiles [62]. Similarly, phthalate exposure has been linked to impaired lipid metabolism and dyslipidemia in both animal and human studies [63, 64].

The observed association between maternal EDC exposure and lower DBP but higher SBP in children suggests that EDCs may exert differential effects on blood pressure regulation depending on various factors, including the specific types of EDCs involved, timing of exposure, and individual susceptibility. This discrepancy underscores the multifaceted nature of EDC-induced alterations in cardiovascular health. One potential mechanism underlying the observed associations involves disruption of the renin–angiotensin–aldosterone system (RAAS), a key regulator of blood pressure and fluid balance. EDCs, such as BPA and phthalates, have been shown to interfere with RAAS signaling pathways through various mechanisms, including activation of angiotensin receptors and modulation of aldosterone synthesis and secretion [65, 66]. Dysregulation of RAAS activity can lead to alterations in vascular tone, sodium retention, and fluid volume, ultimately impacting blood pressure regulation in offspring exposed to EDCs during critical periods of development [67, 68]. Furthermore, emerging evidence suggests that maternal EDC exposure may influence vascular function and endothelial homeostasis, contributing to alterations in blood pressure regulation in offspring. EDCs, including PCBs and organochlorine pesticides, have been shown to impair endothelial function and induce vascular inflammation through oxidative stress-mediated mechanisms [69, 70]. Additionally, EDC-induced alterations in neuroendocrine signaling pathways, such as the sympathetic nervous system and hypothalamic–pituitary–adrenal (HPA) axis, may also contribute to changes in blood pressure regulation in offspring. EDCs, such as phthalates and perfluoroalkyl substances (PFAS), have been implicated in dysregulation of sympathetic nerve activity and cortisol secretion, which can influence vascular tone and blood pressure responsiveness [71, 72].

The association between maternal EDC exposure and child adiposity, as assessed by BMI z-score and waist circumference (WC) z-score, was consistently significant. EDCs, such as BPA and phthalates, have been shown to interfere with endocrine systems involved in adipocyte differentiation, proliferation, and metabolism. These chemicals can disrupt hormone receptors, including peroxisome proliferator-activated receptors (PPARs) and estrogen receptors, leading to dysregulation of adipogenic and lipogenic pathways [1, 73]. For instance, BPA exposure has been associated with increased adipocyte size, altered adipokine secretion, and impaired insulin sensitivity in animal and human studies [74, 75]. Similarly, phthalate exposure has been linked to adipocyte hypertrophy, adipose tissue inflammation, and insulin resistance in both experimental models and epidemiological studies [76, 77].

The present study had some limitations that should be considered in the interpretation of the results. First, heterogeneity between studies was high in most of the evaluated variables. Second, difficulty in discriminating between the effects of exposure during pregnancy and exposure after birth. Third, some of the included studies in the meta-analysis had relatively small sample sizes, which could affect the statistical power to detect significant associations. Fourth, due to the observational nature of the included cohort studies, causal relationships between maternal exposure to EDCs and cardio-metabolic risk factors in children cannot be definitively established.

In conclusion, prenatal exposure to EDCs during the uterine period may elevate the risk of childhood obesity, particularly the visceral form. BPA and pesticides demonstrated the strongest association with WC and BMI z-score. Furthermore, urine sampling from mothers to assess BPA and pesticide concentrations in the first trimester of pregnancy revealed a significant linear association with BMI and WC z-scores in children aged 2–8 years. Therefore, identifying these pollutants and their sources is crucial for preventing childhood obesity. According to our review, no study was founded in Iran about maternal exposure to EDCs during gestation and their consequences on the growth and metabolic markers in children. Based on the differences about environmental contaminants, it is suggested to researchers on Iranian population for future studies. All community members, especially pregnant women, the next generations, policy and health decision makers will benefit from these results. However, the cause-effect of EDCs on metabolism is lacking and precise molecular mechanisms are unclear. More molecular studies are needed in this field.

### Supplementary Information

**Additional file 1: Table S1.** Detailed search strategies of the association between EDCs exposure and risk of EMs. **Table S2.** Quality assessment of studies included in the meta-analysis. **Table S3.** Subgroup analyses for association between maternal exposures to the EDC and serum TG in children. **Fig S1.** Funnel plot for serum triglyceride level. **Fig S2**. Funnel plot for serum total cholesterol in children. **Table S4.** Subgroup analysis of association between maternal exposures to the EDCs with serum HDL-C in children. **Fig S3.** Funnel plot for HDL- C in children. **Fig S4.** Funnel plot for SBP (**A**) and DBP (**B**) in children. **Table S5.** Subgroup analysis of association between maternal exposures to the EDCs and blood pressure in children. **Table S6.** Subgroup analysis of association between maternal exposures to the EDC with BMI and waist circumference z-score in children. **Fig S5.** Funnel plot for BMI and WC z-score in children.

## Data Availability

Data will be available by the corresponding author based on request.
