# Supplementary material for: Association of maternal exposure to endocrine disruptor chemicals with cardio-metabolic risk factors in children during childhood: a systematic review and meta-analysis of cohort studies
Source: Diabetol Metab Syndr. 2024 Apr 4;16:82. doi: 10.1186/s13098-024-01320-0 (PMC10993545; doi:10.1186/s13098-024-01320-0)
Supplement: Supplementary file 1 — Additional file 1: Table S1. Detailed search strategies of the association between EDCs exposure and risk of EMs. Table S2. Quality assessment of studies included in the meta-analysis. Table S3. Subgroup analyses for association between maternal exposures to the EDC and serum TG in children. Fig S1. Funnel plot for serum triglyceride level. Fig S2. Funnel plot for serum total cholesterol in children. Table S4. Subgroup analysis of association between maternal exposures to the EDCs with serum HDL-C in children. Fig S3. Funnel plot for HDL- C in children. Fig S4. Funnel plot for SBP (A) and DBP (B) in children. Table S5. Subgroup analysis of association between maternal exposures to the EDCs and blood pressure in children. Table S6. Subgroup analysis of association between maternal exposures to the EDC with BMI and waist circumference z-score in children. Fig S5. Funnel plot for BMI and WC z-score in children. [file 13098_2024_1320_MOESM1_ESM.docx]

Additional file 1

Table S1: Detailed search strategies of the association between EDCs exposure and risk of EMs.

| Database | Search strategy | |
| --- | --- | --- |
| Pubmed | #1 Endocrine disruptors  #2 Endocrine disrupting chemicals  #3 EDCs  #4 Bisphenol A  #5 BPA  #6 Polychlorinated biphenyls  #7 PCBs  #8 Organochlorine pesticides  #9 OCPs  #10 Phthalate esters  #11 PAEs  #12 Di-(2-ethylhexyl)-phthalate  #13 DEHP  #14 #1 OR #2 OR #3 OR #4 OR #5 OR #6 OR #7 OR #8 OR #9 OR #10 OR #11 OR #12 OR #13  #15 "Lipid Profile"  #16 Triglycerides  #17 TG  #18 LDL  #19 HDL  #20 low density lipoprotein  #21 high density lipoprotein  #22 total cholesterol  #23 weight  #24 "body mass index"  #25 BMI  #26 waist circumference  #27 "blood pressure"  #28 "systolic blood pressure"  #29 "diastolic blood pressure"  #30 #15 OR #16 OR 17 OR 18 OR 19 OR 20 OR 21 OR 22 OR 23 OR 24 OR 25 OR 26 OR 27 OR 28 OR 29  #18 #14 AND #30 | (Endocrine disruptors OR Endocrine disrupting chemicals OR EDCs OR Bisphenol A OR BPA OR Polychlorinated biphenyls OR PCBs OR Organochlorine pesticides OR OCPs OR Phthalate esters OR PAEs OR (Di-(2-ethylhexyl)-phthalate) OR DEHP) AND (hypertension[Title/Abstract]) OR (CVD[Title/Abstract])) OR (overweight[Title/Abstract])) OR (obesity[Title/Abstract])) OR (diabetes[Title/Abstract])) OR ("metabolic syndrome"[Title/Abstract])) OR (dyslipidemia[Title/Abstract])) OR (glycaemia[Title/Abstract])) OR ("fasting blood sugar"[Title/Abstract])) OR (FBS[Title/Abstract])) OR (Insulin[Title/Abstract])) OR (HOMA-IR[Title/Abstract])) OR (QUICKI[Title/Abstract])) OR ("total cholesterol"[Title/Abstract])) OR (triglycerides[Title/Abstract])) OR ("systolic blood pressure"[Title/Abstract])) OR ("diastolic blood pressure"[Title/Abstract])) OR ("LDL-cholesterol"[Title/Abstract])) OR (LDL[Title/Abstract])) OR (" low-density lipoprotein"[Title/Abstract])) OR (" high-density lipoprotein"[Title/Abstract])) OR (HDL[Title/Abstract])) OR (HDL-cholesterol[Title/Abstract])) OR (inflammation[Title/Abstract])) OR ("lipid profile"[Mesh])) OR ("Glucose Homeostasis"[Mesh])) OR ("Metabolic Syndrome X"[Mesh])) OR ("cardiometabolic Syndrome"[Title/Abstract])) OR ("Insulin Resistance Syndrome"[Title/Abstract])) OR ("Metabolic X Syndrome"[Title/Abstract])) OR ("Dysmetabolic Syndrome"[Title/Abstract])) OR ("Cardiovascular Syndromes, Metabolic"[Title/Abstract])) OR ("Diabetes Mellitus, Type 2"[Mesh])) OR ("obesity"[Mesh])) OR (" abdominal obesity"[Mesh])) OR (cardiometabolic[Title/Abstract])) |

Table S2: Quality assessment of studies included in the meta-analysis

|  | **Selection** | | | | **Comparability** | | **Outcome** | | | **Total quality score** |
| --- | --- | --- | --- | --- | --- | --- | --- | --- | --- | --- |
| **Study** | Representativeness of exposed cohort | Selection of non-  exposed cohort | Ascertainment  of exposure | Demonstration that outcome  of interest was not present at start of study | Adjust for the most important  risk factors | Adjust for other  risk factors | Assessment of outcome | Follow-up  length | Loss to  follow-up  rate |  |
| Montazeri 2022 | 1 | 0 | 1 | 1 | 1 | 1 | 1 | 1 | 0 | 7 |
| Zuo 2022 | 1 | 1 | 1 | 1 | 1 | 1 | 1 | 1 | 1 | 9 |
| Berger 2021 | 1 | 1 | 0 | 1 | 1 | 0 | 1 | 1 | 1 | 7 |
| Yang 2021 | 1 | 0 | 1 | 1 | 1 | 0 | 1 | 1 | 1 | 7 |
| Güil-Oumrait 2021 | 1 | 1 | 1 | 0 | 1 | 1 | 1 | 1 | 0 | 7 |
| Jensen 2020 | 0 | 0 | 1 | 1 | 1 | 1 | 1 | 1 | 0 | 6 |
| Kupsco 2020 | 1 | 0 | 1 | 0 | 1 | 0 | 1 | 1 | 0 | 5 |
| Warner 2020 | 1 | 1 | 1 | 1 | 1 | 0 | 1 | 1 | 0 | 7 |
| Sol 2020 | 1 | 1 | 0 | 1 | 1 | 0 | 1 | 1 | 0 | 6 |
| Ouyang 2020 | 1 | 1 | 1 | 1 | 1 | 1 | 1 | 1 | 1 | 9 |
| Warner 2019 | 1 | 1 | 1 | 0 | 1 | 0 | 1 | 1 | 1 | 7 |
| Manzano-Salgado 2017 | 1 | 1 | 1 | 1 | 1 | 1 | 1 | 1 | 0 | 8 |
| Bae 2017 | 1 | 1 | 1 | 0 | 1 | 0 | 1 | 1 | 0 | 6 |
| Vafeiadi 2016 | 0 | 1 | 1 | 0 | 1 | 0 | 1 | 1 | 1 | 6 |
| Braun 2016 | 0 | 0 | 1 | 1 | 1 | 0 | 1 | 1 | 1 | 6 |
| Vafeiadi 2015 | 1 | 1 | 0 | 1 | 1 | 0 | 1 | 1 | 1 | 7 |
| Kim 2015 | 1 | 1 | 0 | 1 | 0 | 1 | 1 | 1 | 0 | 6 |
| Cupul-Uicab 2013 | 1 | 1 | 0 | 0 | 1 | 1 | 1 | 1 | 0 | 6 |
| Smink 2008 | 1 | 0 | 1 | 1 | 1 | 1 | 1 | 1 | 0 | 7 |
| Lee 2019 | 1 | 1 | 1 | 1 | 1 | 0 | 1 | 1 | 1 | 8 |
| Maresca 2016 | 1 | 0 | 1 | 1 | 1 | 1 | 1 | 1 | 1 | 8 |
| Harlely 2013 | 1 | 1 | 0 | 1 | 1 | 1 | 1 | 1 | 1 | 8 |
| Buckley 2016 | 1 | 1 | 1 | 1 | 1 | 1 | 1 | 1 | 1 | 9 |
| Valvi 2015 | 1 | 1 | 0 | 0 | 1 | 1 | 1 | 1 | 1 | 7 |
| Erkin-Cakma 2015 | 1 | 1 | 1 | 1 | 1 | 0 | 1 | 1 | 0 | 7 |
| Agay-Shay K 2014 | 1 | 1 | 1 | 1 | 1 | 1 | 1 | 1 | 1 | 9 |
| Tang-Pe ́ronard 2014 | 1 | 1 | 1 | 1 | 1 | 1 | 1 | 1 | 0 | 8 |
| Warner 2014 | 1 | 1 | 1 | 0 | 1 | 1 | 1 | 1 | 1 | 8 |
| Harlely 2013 | 1 | 0 | 1 | 1 | 1 | 0 | 1 | 1 | 0 | 6 |
| Andersen 2013 | 1 | 1 | 1 | 1 | 1 | 1 | 1 | 1 | 1 | 9 |
| Delvaux 2013 | 1 | 0 | 1 | 0 | 1 | 1 | 1 | 1 | 1 | 7 |
| Lee 2019 | 1 | 1 | 1 | 1 | 1 | 1 | 1 | 1 | 0 | 8 |
| Vuong 2016 | 1 | 1 | 1 | 1 | 1 | 0 | 1 | 1 | 0 | 7 |

**Table S3.** Subgroup analyses for association between maternal exposures to the EDC and serum TG in children

|  | Number of effect sizes | Fisher_Z (95% CI) | P effect |  | I^2^ (%) |  |
| --- | --- | --- | --- | --- | --- | --- |
| Country |  |  |  |  |  |  |
| USA | 1 | -0.27(-0.36, -0.17) | <0.001 |  | 0.0 |  |
| Other countries | 5 | 0.02 (-0.01, 0.06) | 0.231 |  | 97.9 |  |
| Sample type |  |  |  |  |  |  |
| Serum | 3 | 0.1 (0.05, 0.14) | <0.001 |  | 98.1 |  |
| Urine | 3 | -0.25 (-0.31, -0.19) | <0.001 |  | 93.5 |  |
| Pregnancy time for sampling |  |  |  |  |  |  |
| first trimester of pregnancy | 3 | 0.03 (-0.02, 0.07) | 0.254 |  | 97.9 |  |
| Two or three trimester of pregnancy | 3 | -0.07 (-0.12, -0.02) | 0.005 |  | 98.3 |  |
| Child evaluation year |  |  |  |  |  |  |
| ≤4 | 3 | -0.24 (-0.3, -0.19) | <0.001 |  | 93.4 |  |
| >4 | 3 | 0.11 (0.07, 0.15) | <0.001 |  | 0.98 |  |
| EDC Type |  |  |  |  |  |  |
| BPA | 1 | -0.35 (-0.44, -0.27) | <0.001 |  | 0.0 |  |
| Phthalate | 2 | -0.17 (-0.25, -0.09) | <0.001 |  | 95.3 |  |
| Pesticides  Other EDC | 1  2 | 0.38 (0.28, 0.47)  0.03(-0.02, 0.08) | <0.001  0.310 |  | 0.00  98.5 |  |
| Pesticides  Other EDC | 2  0 | -0.3 (-0.36, -0.24) | <0.001 |  | 99 |  |

_USA; United States of America, EDC; endocrine disruptive chemicals, TG; triglyceride, I2; heterogeneity_


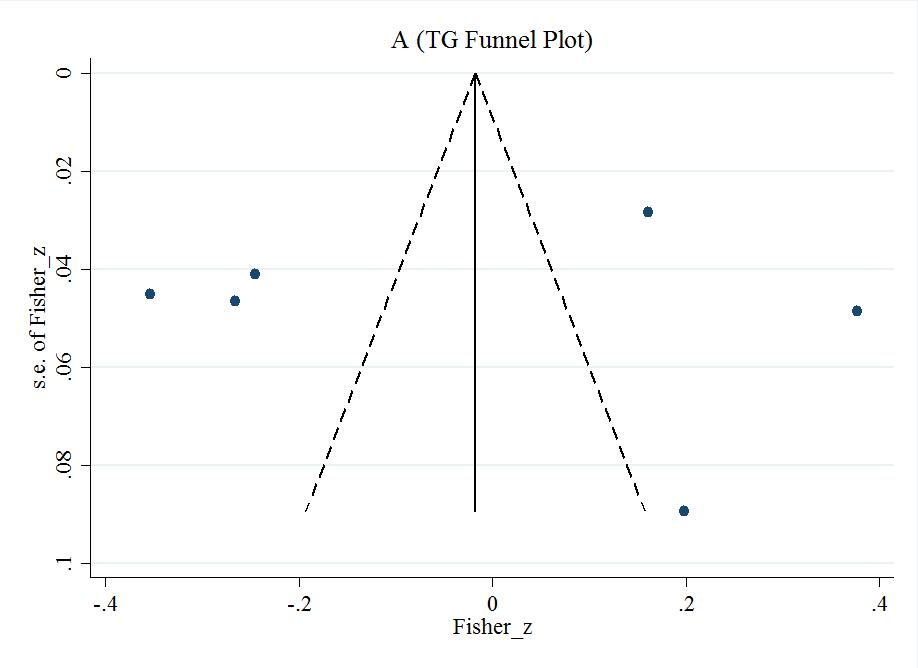


**Fig S1.** Funnel plot for serum triglyceride level


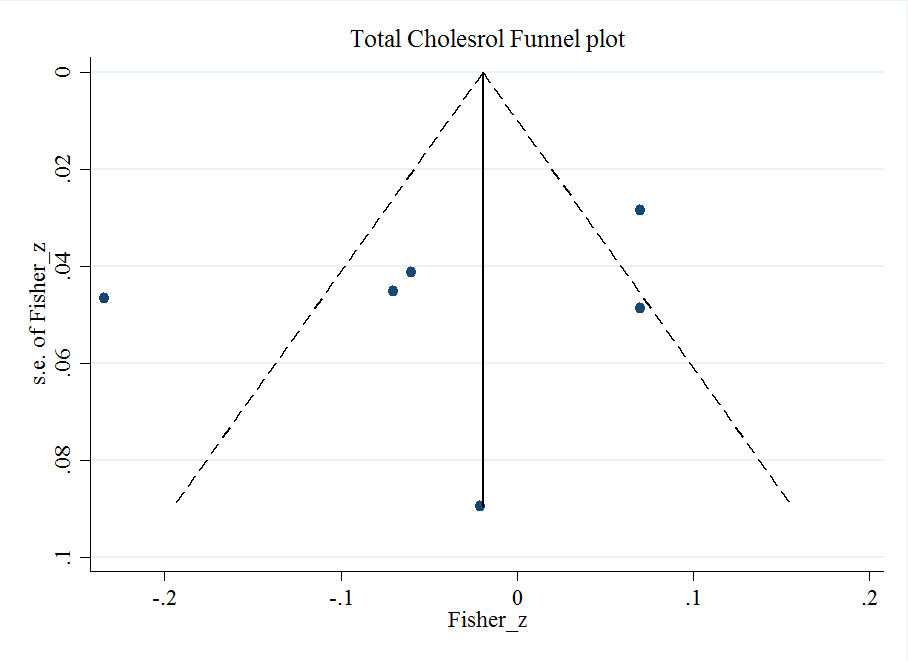


**Fig S2**. Funnel plot for serum total cholesterol in children

**Table S4.** Subgroup analysis of association between maternal exposures to the EDCs with serum HDL-C in children

|  | Number of effect sizes | Fisher_Z (95% CI) | P effect |  | I^2^ (%) |  |
| --- | --- | --- | --- | --- | --- | --- |
| Country |  |  |  |  |  |  |
| USA | 1 | 0.31(0.22, 0.4) | <0.001 |  | 0.0 |  |
| non-USA | 4 | -0.08 (-0.12, -0.04) | <0.001 |  | 98.8 |  |
| Sample type |  |  |  |  |  |  |
| Serum | 3 | 0.05 (0.01, 0.09) | 0.012 |  | 87.4 |  |
| Urine | 2 | -0.21 (-0.27, -0.14) | <0.001 |  | 99.6 |  |
| Pregnancy time for sampling |  |  |  |  |  |  |
| first trimester of pregnancy | 2 | -0.21 (-0.25, -0.16) | <0.001 |  | 99.4 |  |
| Two or three trimester of pregnancy | 3 | 0.19 (0.13,0.24) | <0.001 |  | 86.9 |  |
| Child evaluation year |  |  |  |  |  |  |
| ≤4 | 3 | -0.13 (-0.18, -0.09) | <0.001 |  | 99 |  |
| >4 | 2 | 0.26 (0.2, 0.33) | <0.001 |  | 51.1 |  |
| EDC Type |  |  |  |  |  |  |
| BPA | 1 | -0.69 (-0.78, -0. 6) | <0.001 |  | - |  |
| Phthalate | 1 | 0.31 (0.22, 0.40) | <0.001 |  | - |  |
| Pesticides  Other EDC | 0  3 | -  0.05(0.01,0.09) | -  0.012 |  | -  87.4 |  |

_USA; United States of America, EDC; endocrine disruptive chemicals, HDL-C; high density lipoprotein-cholesterol, I2; heterogeneity_


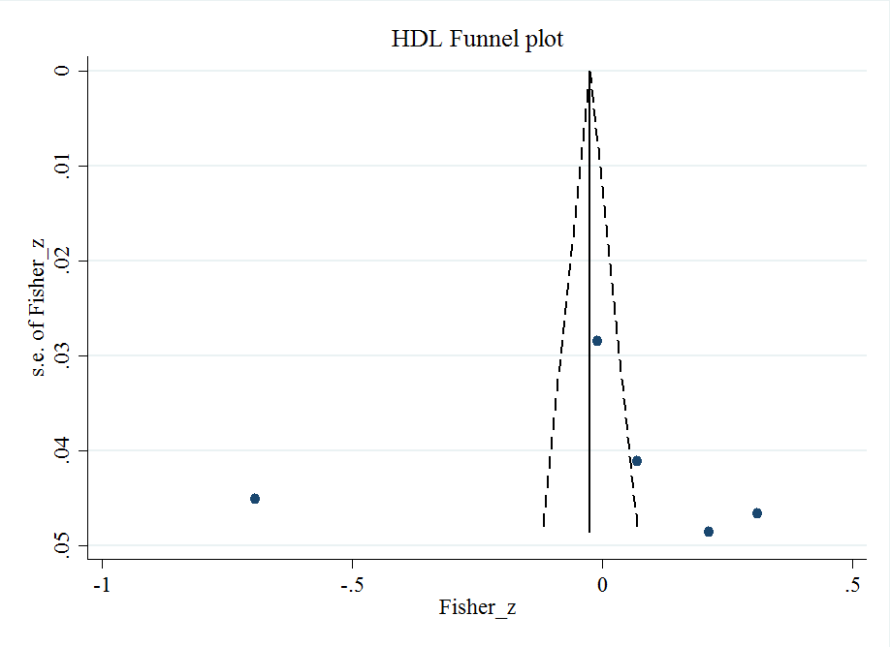


**Fig S3**. Funnel plot for HDL- C in children

**Table S5.** Subgroup analysis of association between maternal exposures to the EDCs and blood pressure in children

|  | Number of effect sizes | Fisher_Z (95% CI) | P effect |  | I^2^ (%) |  |
| --- | --- | --- | --- | --- | --- | --- |
| **DBP** | | | | | | |
| Country |  |  |  |  |  |  |
| USA | 0 | - | - |  | 0.0 |  |
| Others country | 14 | -0.16 (-0.19, -0.13) | <0.001 |  | 98.6 |  |
| Sample type |  |  |  |  |  |  |
| Serum | 1 | 0.12 (0.02, 0.22) | 0.019 |  | 0.0 |  |
| Urine | 13 | -0.54 (-0.61, -0.46) | <0.001 |  | 98.7 |  |
| Pregnancy time for sampling |  |  |  |  |  |  |
| first trimester of pregnancy | 6 | -0.54 (-0.58, -0.49) | <0.001 |  | 98.7 |  |
| Two or three trimester of pregnancy | 8 | 0.05 (0.01, 0.08) | 0.005 |  | 93.5 |  |
| Child evaluation year |  |  |  |  |  |  |
| ≤4 | 6 | -0.25 (-0.29, -0.21) | <0.001 |  | 99.4 |  |
| >4 | 8 | -0.10 (-0.13, -0.07) | <0.001 |  | 94.9 |  |
| EDC Type |  |  |  |  |  | <0.001 |
| BPA | 7 | -0.17 (-0.21, -0.14) | <0.001 |  | 99.2 |  |
| Phthalate | 5 | -0.07 (-0.11, -0.03) | 0.001 |  | 95.3 |  |
| Pesticides  Other EDC | 2  0 | -0.3 (-0.36, -0.24) | <0.001 |  | 99 |  |
| **SBP** | | | | | | |
| Country |  |  |  |  |  |  |
| USA | 0 | - | - |  | - |  |
| Others country | 15 | 0.06 (0.04, 0.08) | <0.001 |  | 94.2 |  |
| Sample type |  |  |  |  |  |  |
| Serum | 2 | 0.04 (-0.01, 0.09) | 0.134 |  | 91.5 |  |
| Urine | 13 | 0.05 (0.02, 0.07) | 0.001 |  | 94.8 |  |
| Pregnancy time for sampling |  |  |  |  |  |  |
| first trimester of pregnancy | 7 | 0.07 (0.03, 0.1) | <0.001 |  | 97.1 |  |
| Two or three trimester of pregnancy | 8 | 0.05 (0.02, 0.09) | <0.0.001 |  | 80.2 |  |
| Child evaluation year |  |  |  |  |  |  |
| ≤4 | 7 | 0.07 (0.04, 0.1) | <0.001 |  | 92.5 |  |
| >4 | 8 | 0.05 (0.02, 0.09) | 0.002 |  | 95.7 |  |
| EDC Type |  |  |  |  |  |  |
| BPA | 2 | -10.80 (-52.47, 30.87) | 0.611 | <0.001 | 96 |  |
| Phthalate | 2 | -10.87 (-25.99, 4.26) | 0.159 | 0.714 | 0.0 |  |
| Pesticides  Other EDC | 2 | -8.20 (-29.79, 13.38) | 0.456 | 0.063 | 71.1 |  |

_USA; United States of America, EDC; endocrine disruptive chemicals, DB; diastolic blood pressure, SBP; systolic blood pressure, I2; heterogeneity_


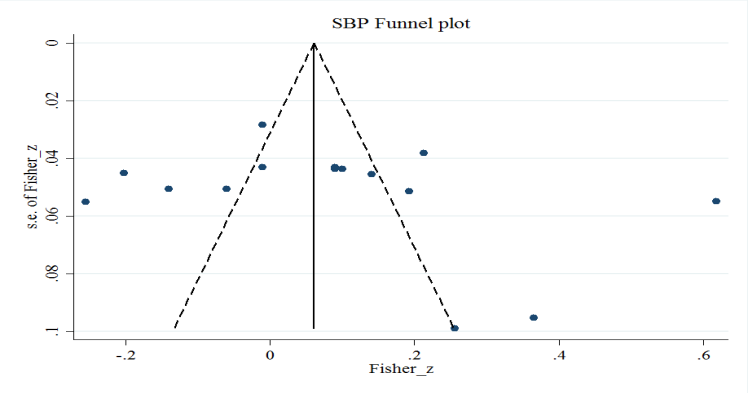

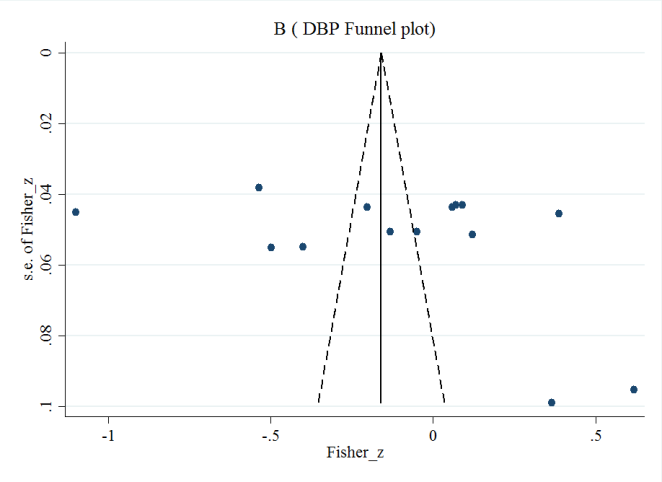


1. diastolic
2. systolic

**Fig S4.** Funnel plot for SBP (A) and DBP (B) in children

**Table S6.** Subgroup analysis of association between maternal exposures to the EDC with BMI and waist circumference z-score in children

|  | Number of effect sizes | Fisher_Z (95% CI) | P effect |  | I^2^ (%) |  |
| --- | --- | --- | --- | --- | --- | --- |
| **BMI Z-score** | | | | | | |
| Country |  |  |  |  |  |  |
| USA | 11 | -0.01(-0.03, 0.02) | 0.732 |  | 89.9 |  |
| Other countries | 18 | 0.07 (0.05, 0.09) | <0.001 |  | 91.7 |  |
| Sample type |  |  |  |  |  |  |
| Serum | 15 | 0.01 (-0.01, 0.03) | 0.382 |  | 91.7 |  |
| Urine | 14 | 0.09 (0.06, 0.12) | <0.001 |  | 90.1 |  |
| Pregnancy time for sampling |  |  |  |  |  |  |
| first trimester of pregnancy | 10 | 0.09 (0.07, 0.12) | <0.001 |  | 91.2 |  |
| Two or three trimester of pregnancy | 19 | -0.00 (-0.02, 0.02) | 0.969 |  | 90.5 |  |
| Child evaluation year |  |  |  |  |  |  |
| ≤4 | 8 | 0.08 (0.05, 0.11) | <0.001 |  | 89.3 |  |
| >4 | 21 | 0.02 (-0.00, 0.04) | 0.065 |  | 91.8 |  |
|  |  |  |  |  |  |  |
| EDC Type |  |  |  |  |  |  |
| BPA  Phthalate  Pesticides  Other EDC | 3  9  11  6 | 0.14(0.08, 0.19)  0.01 (-0.03, 0.04)  0.1(0.08, 0.12)  -0.09(-0.13,-0.06) | <0.001  0.734  <0.001  <0.001 |  | 91.7  82.3  90.3  91.5 |  |
| **waist circumference Z-score** | | | | | | |
| Country |  |  |  |  |  |  |
| USA | 7 | 0.16 (0.11, 0.20) | <0.001 |  | 99.6 |  |
| Other countries | 12 | 0.02 (-0.00, 0.05) | 0.103 |  | 86.7 |  |
| Sample type |  |  |  |  |  |  |
| serum | 13 | 0.01 (-0.02, 0.03) | 0.72 |  | 98.2 |  |
| urine | 6 | 0.19 (0.15, 0.24) | <0.001 |  | 99.6 |  |
| Pregnancy time for sampling |  |  |  |  |  |  |
| first trimester of pregnancy | 6 | 0.14 (0.10, 0.17) | <0.001 |  | 99.6 |  |
| Two or three trimester of pregnancy | 23 | 0.01 (-0.02, 0.03) | 0.399 |  | 98.1 |  |
| Child evaluation year |  |  |  |  |  |  |
| ≤4 | 3 | -0.03 (-0.06, 0.01) | 0.118 |  | 88.1 |  |
| >4 | 3 | 0.12 (0.09, 0.15) | <0.001 |  | 99.3 |  |
| EDC Type |  |  |  |  |  |  |
| BPA | 1 | 1.59 (1.47, 1.70) | <0.001 |  | - |  |
| Phthalate | 5 | 0.03 (-0.02, 0.08) | 0.289 |  | 99.1 |  |
| Pesticides  Other EDC | 7  6 | 0.08 (0.04, 0.12)  -0.11(-0.15, -0.07) | <0.001  <0.001 |  | 97.2  98.8 |  |

_USA; United States of America, EDC; endocrine disruptive chemicals, BMI; body mass index, I2; heterogeneity_


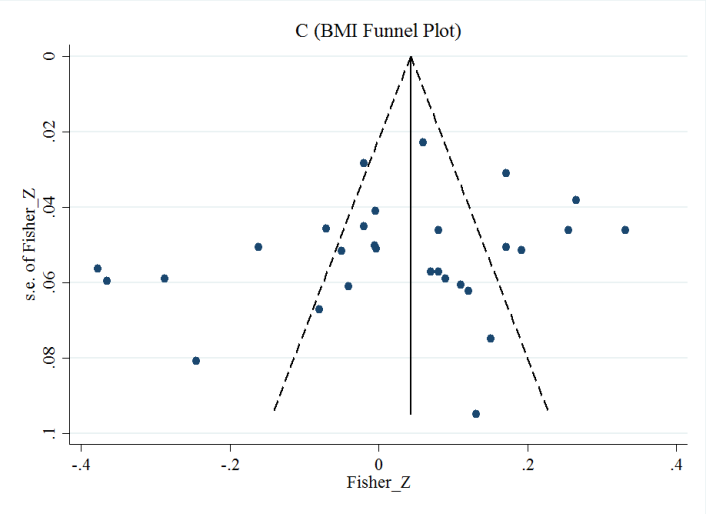

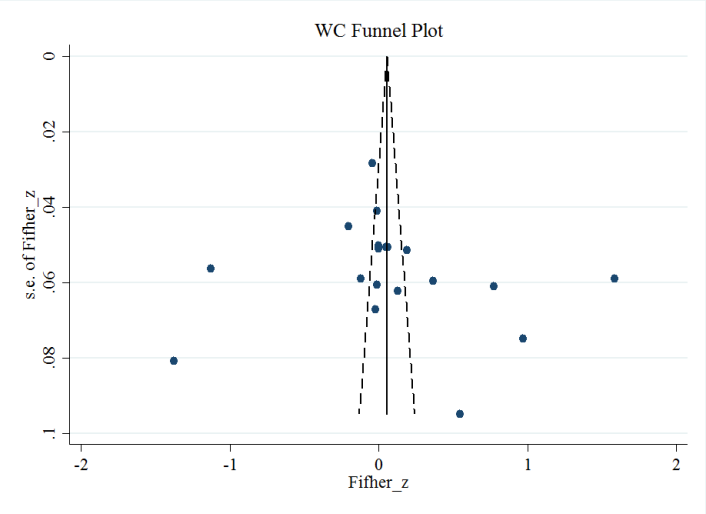


**Fig S5.** Funnel plot for BMI and WC z-score in children
